# Social and emotional wellbeing of Aboriginal and Torres Strait Islander peoples in Aboriginal controlled social housing

**DOI:** 10.1186/s12889-023-16817-y

**Published:** 2023-10-06

**Authors:** Alison Brown, Tilahun Haregu, Graham Gee, Fiona Mensah, Lea Waters, Stephanie J Brown, Jan M Nicholson, Kelsey Hegarty, Darren Smith, Sue D’Amico, Rebecca Ritte, Yin Paradies, Gregory Armstrong

**Affiliations:** 1 The University of Melbourne, Parkville, VIC 3010 Australia; 2 Murdoch Children’s Research Institute, The Royal Children’s Hospital, Parkville, VIC 3052 Australia; 3 School of Psychological Sciences, University of Melbourne, Parkville, VIC 3010 Australia; 4 Latrobe University, Bundoora, VIC 3086 Australia; 5 The Royal Women’s Hospital, Parkville, VIC 3052 Australia; 6 Aboriginal Housing Victoria, Fitzroy North, VIC 3068 Australia; 7 Deakin University, Burwood, VIC 3125 Australia

**Keywords:** Aboriginal and Torres Strait Islander, Social housing, Social and emotional wellbeing, Needs and aspirations

## Abstract

**Background:**

Little is known about the wellbeing and aspirations of Aboriginal and Torres Strait Islander peoples living in social housing. Aboriginal and Torres Strait Islander peoples living in social housing face common social housing challenges of low income, higher incidence of mental health issues and poorer health along with specific challenges due to the impacts of colonisation and its ongoing manifestations in racism and inequity. A greater understanding of social and emotional wellbeing needs and aspirations is essential in informing the provision of appropriate support.

**Methods:**

Surveys of social and emotional wellbeing (SEWB) were completed by 95 Aboriginal people aged 16 years and older living in Aboriginal Housing Victoria social housing in 2021. The survey addressed a range of domains reflecting social and emotional wellbeing, as defined by Aboriginal and Torres Strait Islander peoples.

**Results:**

Most respondents demonstrated a strong sense of identity and connection to family however 26% reported having 6 or more health conditions. Ill health and disability were reported to be employment barriers for almost a third of people (32%). Improving health and wellbeing (78%) was the most cited aspiration. Experiences of racism and ill health influenced engagement with organisations and correspondingly education and employment.

**Conclusion:**

Strong connections to identity, family and culture in Aboriginal peoples living in social housing coexist along with disrupted connections to mind, body and community. Culturally safe and appropriate pathways to community services and facilities can enhance these connections. Research aimed at evaluating the impact of strengths-based interventions that focus on existing strong connections will be important in understanding whether this approach is effective in improving SEWB in this population.

**Trial Registration:**

: This trial was retrospectively registered with the ISRCTN Register on the 12/7/21 with the study ID:ISRCTN33665735.

**Supplementary Information:**

The online version contains supplementary material available at 10.1186/s12889-023-16817-y.

## Background

Secure affordable housing is fundamental to providing stability and security for individuals and families [1]. In Australia, social housing is provided by government (public housing) or community sector organisations (community housing) to people on low incomes, especially those at risk of homelessness or family violence. Due to limited housing stock, social housing residents are prioritised according to need [2]. Residents may experience a range of stressors such as unemployment, poor physical or mental health or disability which can impact their wellbeing [2, 3]. Aboriginal and Torres Strait Islander peoples living in social housing face additional challenges and stressors due to the impacts of colonisation and dislocation, and their ongoing manifestations in racism and inequity [4, 5]. The complex and multiple needs of some Aboriginal and Torres Strait Islander peoples are evident in high rates of mental and physical health issues, social exclusion, trauma, family breakdown, psychological distress and personal safety fears [4, 6–8].

A body of literature exists on the wellbeing and needs of social housing tenants, however, little is known about the wellbeing, needs and aspirations of Aboriginal and Torres Strait Islander peoples living in social housing. Freund et al. [9] in an exploratory study of wellbeing needs of people living in social housing found that those who identified as Aboriginal and Torres Strait Islander had a higher number of wellbeing needs than those who were non-Indigenous.

**Fig. 1:**
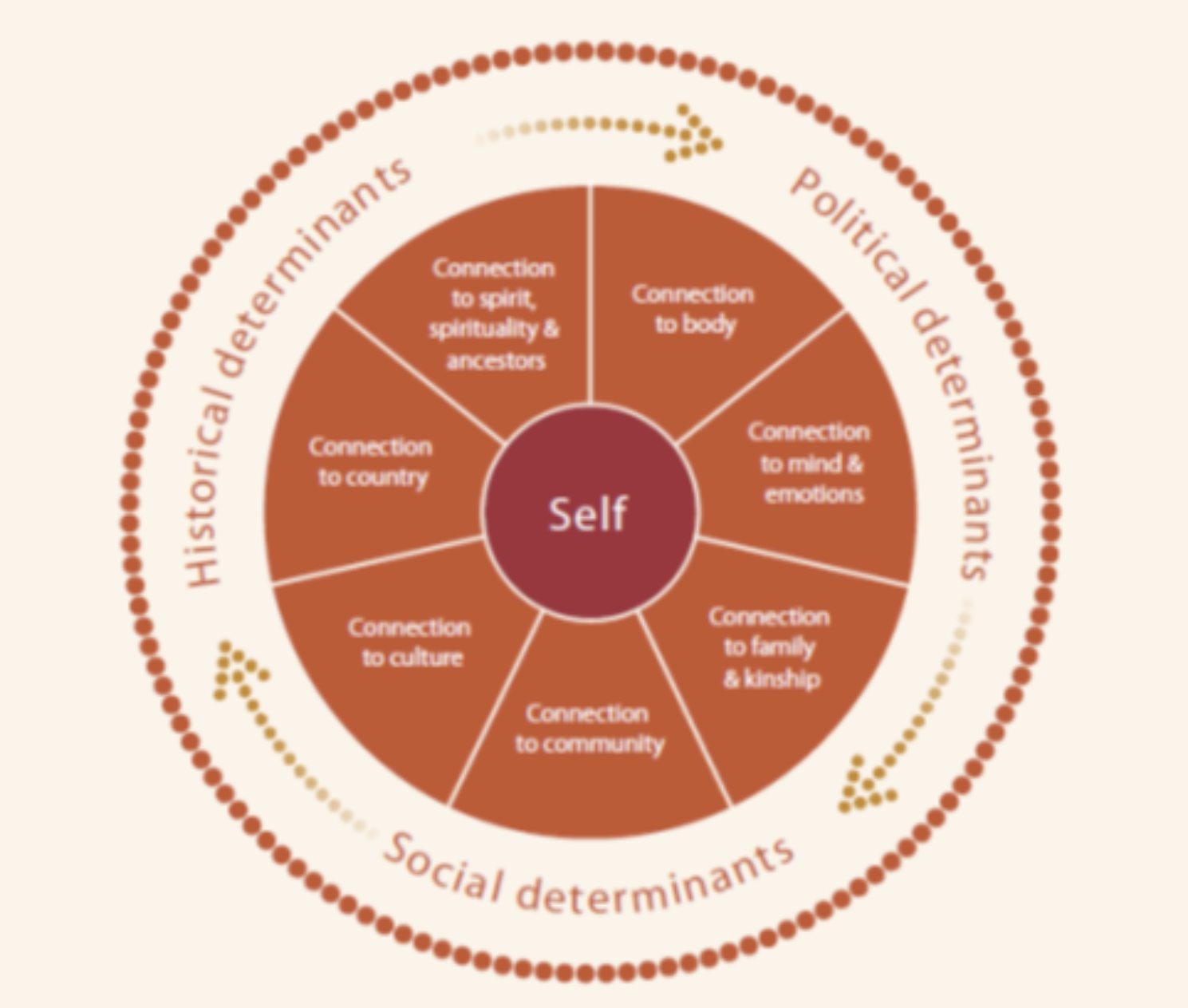
A model of Social and Emotional Wellbeing. Reprinted with permission from National Strategic Framework for Aboriginal and Torres Strait Islander Peoples’ Mental Health and Social and Emotional Wellbeing 2017-23 by Gee, Dudgeon, Schultz, Hart and Kelly, 2013 [12]

Wellbeing for Aboriginal and Torres Strait Islander peoples can be described by the term social and emotional wellbeing (SEWB). In this context, the term refers to multiple elements that are connected and in balance at the individual, family and community levels to contribute to wellbeing [4, 6, 10]. While communities may differ in their interpretation of SEWB, Gee et al. [11] have identified seven common elements including connection to body; mind and emotions; family and kinship; community; culture; country; and, spirituality and ancestors (see Fig. 1). This SEWB framework recognises the role of broader social determinants in shaping SEWB, such as income, employment, housing, education and access to community resources [6, 11], in addition to less recognised historical and political determinants. The latter determinants refer to the degree to which individuals, families, and whole communities/cultural groups have been able to maintain connections to land, culture, community control and self-determination, despite past and ongoing impacts of colonisation [11]. The conceptualisation of self, in this model, views the self as ‘inseparable from, and embedded within, family and community’ [11, p. 57). This understanding informs a framework to holistically understand the SEWB of Aboriginal and Torres Strait Islander peoples who live in social housing.

Identifying needs is important in planning and advocating for appropriate support services. However, it is not enough by itself. Needs can be thought of as changeable, contingent on external conditions and sometimes requiring immediate relief, whereas aspirations are aimed at self-determined positive life changes that are intrinsically motivated [13]. Aspirations provide insight into persistent desires for improving one’s current situation [13]. Hart views aspirations as the ‘kernels or precursors of many important capabilities which support human flourishing’ [14, p. 336). Understanding the aspirations of communities can inform social change, policy, action and investment [14].

This study aims to:

- understand the social and emotional wellbeing, strengths, needs and aspirations of Aboriginal and Torres Strait Islander peoples living in Aboriginal Housing Victoria (AHV) social housing.
- assess the characteristics of respondents to determine the representativeness of the sample in comparison to the broader AHV population.

The findings of this study will inform the implementation of a novel wellbeing intervention by AHV [15].

## Methods

### Study design

We conducted a survey of people living in social housing in three regions provided by AHV. AHV is an Aboriginal community-controlled organisation providing community housing to Aboriginal and Torres Strait Islander peoples living in Victoria, Australia. In addition to core tenancy management activities, AHV provides a range of individual supports to assist a person in maintaining their tenancy along with individual and community support activities that contribute to wellbeing. Their vision is to provide appropriate, affordable housing as a pathway to better lives and stronger communities [16].

The cross-sectional survey assessed social and emotional wellbeing, strengths, needs and aspirations, and served as a tool for identifying people interested in participating in wellbeing support provided by AHV. Peer researchers conducted the survey between April and December 2021, among a voluntary response sample, using a structured questionnaire. The authors used linked administrative data related to tenancies to compare respondents with non-participants.

### Ethics

The study adhered to principles of culturally safe and ethical approaches to research with Aboriginal and Torres Strait Islander communities [17, 18]. Aboriginal researchers were investigators on the research team and Aboriginal advisory group members were involved with leading the project design. The study responded to a community led initiative, involved building the capabilities of local communities through the employment of peer researchers and utilised the knowledge of SEWB as defined by Aboriginal and Torres Strait Islander peoples. This approach reflects a decolonising research method with self-determination and empowerment integral to all aspects of the study including development and data collection [19].

This survey was part of a larger study investigating the implementation of a coaching intervention that received ethics approval from the University of Melbourne Human Ethics Committee STEM 1 on 9/2/21 (Ethics ID 2020-13595-13162-4). Peer researchers assured participants that survey data were held by university researchers and were not available to AHV staff. They also provided interested participants with a plain language statement, explained the research, and documented informed consent via the first survey question. Peer researchers provided a debriefing statement containing information about available support as some participants may be vulnerable to distress due to potential underlying mental health and/or experiences of complex or intergenerational trauma [20]. Survey participants received a $40 voucher for participating in the survey.

### Survey

The survey included domains of social and emotional wellbeing relevant to Aboriginal and Torres Strait Islander peoples. Initially, the survey was used as part of a First 1000 days Australia initiative to describe the aspirations and needs of Aboriginal and Torres Strait Islander families [21]. We modified the survey to shift the focus from parenting to include additional aspects of SEWB and trialed it with 22 Aboriginal and/or Torres Strait community members in the eastern region of Melbourne. We then shortened the survey to reduce the response burden and piloted it with five Aboriginal and Torres Strait Islander. After making further amendments based on the pilot, we finalised the survey.

The survey consisted of demographic information and questions in 12 domains, as outlined in Table 1, and took between 45 and 60 min to complete. We drew survey items from established instruments, modified instruments or instruments developed by researchers or through consultation with AHV or other key stakeholders. We included only questions on psychological and emotional wellbeing from the Mental Health Continuum Short form tool [22] as questions relating to social wellbeing were not considered culturally appropriate by those reviewing the survey. The Aboriginal Resilience and Recovery Questionnaire (ARRQ) has been developed and is validated for use in the Victorian Aboriginal and Torres Strait Islander population [23, 24].

**Table 1:** Survey domains

Survey Domain	Key measures
1. Health	Health Conditions, Euroqual 5D -5 L [25], disability, smoking
2. Wellbeing	Kessler-5 [26], Mental Health Continuum Short Form [22], Aboriginal Resilience and Recovery Questionnaire 25 Items [23, 24]
3. Family	Number of children birthed/adopted/cared for, dependents/age/relationship, Strengths-based parenting tool [27]
4. Culture and Community	Connection to culture and community, community attitudes
5. Self determination	Choice and control in decision making on issues
6. Education, Employment & Finances	Highest level of education completed, employment status, barriers to employment, spending, bill payments, bill stress
7. Service Use	Service use and availability
8. Out of Home Care	Removals/child protection services, age of housing independence, cultural placement plan
9. Cultural safety	Unfair treatment, unsafe environments
10. Housing	Number and type of occupants, household amenities, financial stress due to tenancy
11. Family violence	Experience of controlling, unsafe or threatening behaviour within the family [28]
12. Aspirations	Identification of key categories of goals/aspirations

### Administrative data

AHV routinely collects administrative data on all people who live in social housing in all AHV regions. We linked administrative data to survey data via a unique identifier. We used the administrative data for analyses as well as to compare the characteristics of our survey respondents with the broader AHV social housing population. Administrative data include information on gender, household composition, housing type and a range of data that are indicative of people experiencing difficulties in life including rent in arrears, rent related maintenance charges and complaints (see Table 2).

**Table 2:** Administrative data collection

Domain	Description
Demographic	Gender
Household type	Single, single with children, single with others, couple, couple with children
Housing	Unit or house
Rent in Arrears	Rent in arrears at time point (Yes/No)
Complaints	Number of complaints against household in last 3 months
Tenancy related maintenance charges (TRMC)	Number of TRMC in last 3 months

### Eligibility

Peer researchers invited all Aboriginal and Torres Strait Islander peoples aged 16 years and over, who were living in AHV social housing in three nominated regions (Northwest metropolitan Melbourne, Greater Geelong and Ballarat) to participate in the survey via the main tenancy holder. AHV chose the three regions as a wellbeing program was being trialed in these regions.

### Recruitment and survey administration

AHV employed five Aboriginal and Torres Strait Islander people and one non – Indigenous person, including some who lived in AHV social housing, as peer researchers from local communities to recruit people to the survey. Involving peers in recruitment was important in facilitating engagement with local communities and strengthening community capacity through skill development. Successful applicants underwent a one-week training program in research, ethics and consent, confidentiality, safety, and survey conduct, as well as ongoing supervision from a team leader.

Peer researchers attempted phone contact with the main tenancy holder listed on the tenancy agreement (n = 410 households) in the three regions. They made three attempts to contact all households. They also asked the main tenancy holder if any other people aged 16 and over living at the property might like to participate in the survey.

Peer researcher initially made face to face appointments with consenting participants to explain the survey and provide the participant with a laptop to self-administer the survey. After multiple lockdowns and social restrictions due to the COVID-19 pandemic prevented face to face visits, most surveys were self-administered via an SMS link to a mobile phone version of the electronic survey. Peer researcher survey administration was available over the phone according to the preferences of the participant or the needs of those with low vision, literacy, access to internet or digital skills.

We collected survey data using Qualtrics and exported data to STATA for analysis by researchers at the University of Melbourne.

### Analysis

We analysed survey data in STATA and used descriptive statistics to present the characteristics and SEWB of participants. We collapsed response options to several scales to simplify reporting. For example, we aggregated responses to items in the Mental Health Continuum into the percentage who reported a feeling either ‘2–3 times a week’, ‘almost everyday’ or ‘everyday’. We also aggregated responses for the Aboriginal Resilience and Recovery Questionnaire, self-determination, strength-based parenting, aspirations, and community attitudes questions and this is indicated in the results. Reporting on all responses to questions can be found in Data Supplementary File 2. We compared survey respondents with the full population of Aboriginal and Torres Strait Islander people living in AHV social housing using the administrative data to determine representativeness.

## Results

### Response rate and respondent characteristics

**Fig. 2:**
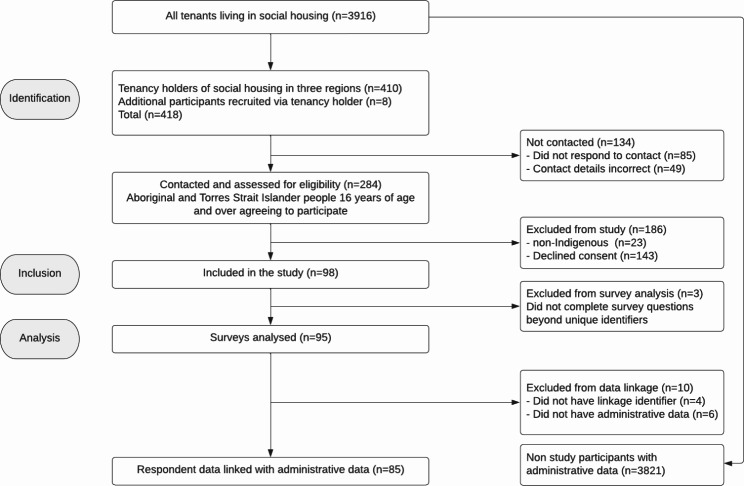
STROBE flow chart. STROBE, Strengthening the Reporting of Observational Studies in Epidemiology

We attempted to contact the tenancy holder for each of the 410 AHV properties in the regions. Tenancy holders from 90 properties participated in the survey, yielding an initial response rate of 22%, with an additional 8 people housed in these properties recruited via the tenancy holder. However, only 255 of the 410 tenancy holders were eligible or able to be contacted by peer researchers with 23 main tenants ineligible due to non-Indigenous status, 49 main tenants having wrong or disconnected contact numbers and 85 not responding to any contact made by peer researchers. The final response rate is therefore 35% of invited tenancy holders. Three respondents did not provide sufficient data beyond basic demographics and were excluded from the sample. The final sample size for survey analysis was 95 respondents (see flow chart in Fig. 2).

All survey respondents identified as Aboriginal (see Table 3).^1^ Most respondents had lived in their current properties for six years or more (57.9%).

**Table 3:** Sample characteristics (n = 95)**

Characteristic	Number	Percent
Gender		
Female	67	70
Male	28	30
**Identity**		
Aboriginal	95	100
**Age**		
16–24	15	16
24–34	11	12
35–44	21	22
45–54	20	21
55–64	12	13
65–74	11	12
75 and over	5	5
**Highest education level**		
Did not attend or complete primary school	*	*
Finished primary school	5	5
Some high school	15	16
Completed year 10 or 11	24	25
Complete high school	10	11
TAFE certificate or Diploma	26	27
University degree or higher	9	10
**Employment status**		
In paid employment (full or part-time, casual and cash in hand work)	17	18
Out of work	16	17
Retired	14	15
Home duties	11	12
Studying	8	8
Unable to work	23	24

*=Cells are < 5 respondents are masked

**Sample size varies per characteristic (ranges 89–95) due to missing data

### Comparison of respondents’ characteristics against the broader AHV population

We compared housing administrative data between survey respondents (n = 85, as administrative data were not available for 6 respondents and 4 respondents did not have linkage identifiers) and the broader AHV population (n = 3,821) to provide further information on representativeness (see data supplementary file 1). The analysis revealed that survey respondents are similar to the broader AHV population on a number of dimensions except in relation to age, gender and household type where they vary significantly. Females were overrepresented in the sample (70%) compared to the broader AHV population of people living in social housing (59%)(p = 0.036). Survey respondents were significantly older with a mean age of 46 years compared with non-survey participants mean of 30 years (p < 0.0001). It was not possible to compare survey respondents with non-participants 16 years old and over as age was not recorded for some in the administrative data. In relation to household type, more than a third (34%) of survey respondents were single as compared to only a fifth (21%) of non-survey participants (p = 0.002). More survey respondents than non-survey participants were single people living in shared houses (p = 0.034). On the other hand, close to 60% of the non-survey participants were in a couple compared to 40% of the survey respondents (p < 0.001). To summarise, the sample was more likely to be female, older, single and living in shared housing compared to non-survey respondents. Importantly, survey respondents and non-survey participants did not significantly vary in terms of the proportion experiencing rent in arrears, tenancy related maintenance charges incurred, complaints made against them and housing type.

### Connection to community, culture, family, and country

The survey included questions on personal, community, relationship and cultural strengths and resources and the results are presented in Table 4. Survey respondents exhibited a strong sense of identity with 80% indicating that they were proud to be Aboriginal or Torres Strait Islander and 77% indicating that being Aboriginal or Torres Strait Islander was an important part of who they are (either ‘a lot’ or ‘a fair bit’). Most respondents (86%) could identify their traditional country or homeland. Similarly, 80% of respondents had knowledge of their mob or mobs (e.g., clan group affiliations). Almost all respondents had been involved in some sort of cultural or community ceremony or event in the last 12 months (97%). Approximately a third of respondents (32%) indicated that they did not participate as often as they wanted in cultural and community celebrations and events due to a range of factors including affordability (33%), being too far away (27%) or a range of commitments preventing involvement (20%).

**Table 4:** Personal, community, relationship and cultural strengths and resources survey results

Survey Questions	Number	Percent
**ARRQ – 25 items** (Response options: not at all/a little/somewhat/a fair bit/a lot)	(Responded a fair bit/a lot)	
I am able to maintain my Aboriginal or Torres Strait Islander identity, values and beliefs	63	66
I feel supported by my friends/mob	57	60
In my community I have opportunities to develop skills (e.g., job skills or skills to care for others)	43	45
I can trust myself to make the right choice	61	64
Being Aboriginal or Torres Strait Islander is an important part of who I am	73	77
I have family that love me even when I muck up	75	79
Spirituality is a source of strength for me	63	66
I am able to deal with most problems that occur in my life	64	67
I have opportunities to work in my life, keep busy and stay involved	50	53
There are people in my life that I have close, secure relationships with	61	64
I am proud to be Aboriginal or Torres Strait Islander	76	80
When changes occur in my life I can usually find ways to adapt	59	62
In my community I have opportunities to further my education	46	48
I can turn to my partner or someone close to me for support and understanding	55	58
I am able to have a laugh even when things are difficult	57	60
I take positive action to try and solve problems	59	62
I participate in cultural practices that give me peace (such as going out bush, ceremony, community cultural events)	39	41
I feel safe when I am with my partner or those closest to me	69	73
I find it easy to get along well with people	59	62
Overall, I feel like I have control over my life	54	57
I have the skills to be confident in both Indigenous and non-Indigenous communities	56	59
I feel safe when I am with my family	71	75
I feel confident in socialising with others around me	51	54
I feel content with my life	61	64
I speak an Aboriginal or Torres Strait Islander language(s)	17	18
Strength based parenting knowledge score n = 38 (Response options strongly agree/agree/neither agree nor disagree/disagree/strongly disagree)	(Responded agree/strongly agree)	
I know the things my kids are good at doing	29	76
I am aware of the strengths my kids have	31	82
I show my kids how to use their strengths in different situations	30	79
I give my kids lots of opportunities to use their strengths	30	79
General survey questions		
I know where my traditional country or homeland is (yes)	82	86
Do you know who your mob/mobs are? (yes)	76	80
Involvement in Ceremony/cultural activities in last 12 months (yes)	93	97
Don’t Participate in cultural activities as often as want due to (n = 30)		
• Can’t afford to • Too far away • Access to the knowledge holders • Caring commitments • Work commitments	10 8 * * *	33 27 * * *
In what environment do you feel culturally safe? • At home with family • With other Aboriginal and / or Torres Strait Islander people	75 43	79 45
Interest in family reconnection services		
Family Tracing Reunion Services Counselling	28 10 10	30 11 11
Removed from family and/or child protection services (yes)	16	17
Relatives removed from family by government (yes)	39	41

*=Cells are < 5 respondents are masked

Family was a source of strength for most people with respondents indicating they had a family that loved them even when they muck up (79%) and felt safe with their family (75%) or their partner or people close to them (73%), either ‘a lot’ or ‘a fair bit’. When asked where they felt most comfortable, respondents reported feeling most comfortable at home with their family (79%) or with other Aboriginal and Torres Strait Islander people (45%). Some respondents expressed interest in family tracing services (30%) and in reunion services (11%) reflecting that 17% of people had been removed from their family and/or placed under care of child protection services and 41% had relatives removed from family by the government.

Most respondents felt content with their life (64%), felt able to make the right choice (64%), were able to deal with problems that occurred (67%) and could adapt to changes (62%), either ’a lot’ or ‘a fair bit’. Just over half felt in control of their lives (57%) either ‘a lot’ or ‘a fair bit’. Most respondents who were parents demonstrated awareness of strength-based parenting skills with 76% agreeing or strongly agreeing with knowing the things their kids were good at, 82% being aware of their kids’ strengths, 79% showing their kids how to use strengths in different situations and 79% giving kids many opportunities to use their strengths.

### Connection to body, mind, and emotions

**Fig. 3:**
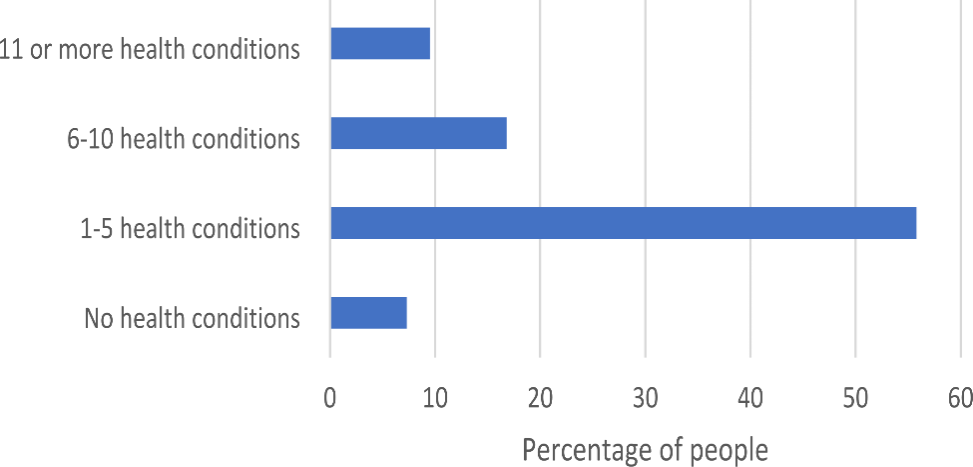
Number of self-reported health conditions as advised by a doctor

The survey explored respondents’ connection to mind, body, and emotions through questions regarding mental and physical health, and the results are presented in Table 5; Fig. 3. Most respondents reported feeling happy (73%), interested in life (71%) and satisfied with life (63%) either a ‘few times a week’, ‘everyday’ or ‘almost everyday’ in the past month. Most respondents also demonstrated psychological wellbeing by liking their personality (70%), managing their responsibilities (78%), having warm and trusting relationships (68%), feeling confident in expressing their own ideas and opinions (72%), having opportunities to grow (67%) and feeling that their life has a sense of direction or purpose (67%) at least a few times a week in the past month. However, 28% reported high or very high levels of psychological distress as measured by the Kessler 5. Eleven % of respondents felt unsafe or afraid in the last 12 months with 8% threatened with physical harm, 7% feeling controlled or put down and 2% subject to physical harm.

**Table 5:** Mental Health and Family Violence

Questions	Number	Percent
**Mental Health Continuum Short form (22)** (Response options: Never/once or twice/about once a week/2–3 times a week/almost everyday/everyday)	(Responded 2–3 times a week/almost everyday/everyday)	
Emotional wellbeing**-** During the past month, how often did you feel:		
Happy	69	73
Interested in life	67	71
Satisfied with life	60	63
Psychological wellbeing**-** During the past month, how often did you feel:		
That you liked most parts of your personality	66	70
Good at managing the responsibilities of your daily life	74	78
That you had warm and trusting relationships with others	65	68
That you had experiences that challenged you to grow and become a better person	64	67
Confident to think or express your own ideas and opinions	68	72
That your life has a sense of direction or meaning to it	64	67
**Kessler 5 score (range 0–25)**(26)	(median = 10)	
Those experiencing high/very high psychological distress (score 12–25)	27	28
Those experiencing moderate/low psychological distress (score 5–11)	62	65
**Family violence questions** (28). In the last 12 months has anyone in your family:		
Made you or your children feel unsafe or afraid?	10	11
Controlled your day-to-day activities or put you down?	7	7
Threatened to hurt you in any way?	8	8
Hit, slapped, kicked or otherwise physically hurt you?	*	*

*=Cells are < 5 respondents are masked

Respondents rated their health out of a hundred on a visual analogue scale and the median was 70 (with 0 representing the worst possible health). 26% of respondents reported having six or more health conditions confirmed by a healthcare provider s (see Fig. 3). The most frequently cited health conditions were anxiety (41%), back problems (36%), asthma (36%), depression (33%) and problems with weight (30%). 61% of respondents noted problems with pain, with 38% reporting chronic pain that is always there or keeps coming back (see supplementary file 2, Health section).

Most respondents (52%) indicated that they or someone in their house had a disability, but awareness of the National Disability Insurance Scheme (NDIS) was low (45%) and only 22% indicated that the person with a disability was receiving NDIS support.

### Employment, education and finances

Survey respondents were asked about social determinants of SEWB through questions related to their employment status, educational achievement and financial situation and the results are presented in Tables 3 and 6. Almost half the survey respondents (45%) had completed either a year 12 or a Certificate III qualification or above. 18% of respondents reported being in some sort of paid employment, with 24% indicating they were unable to work and 18% indicating they were out of work and looking for work (see Table 5). The main barriers to employment were ill health and disability (32%) followed by transport problems (13%) with insufficient skills and no job available, both being cited by 7% of respondents. Less than half the respondents felt they had opportunities to develop skills in the community (45%) or further their education (48%) and just over half of the respondents felt they had opportunities to work in their life, keep busy and stay involved (53%) either ‘a lot’ or ‘a fair bit’ (see ARRQ results in Table 3).

Almost half the respondents (47%) stated they did not have enough money to spend on everyday things. Most respondents (61%) had experienced problems paying bills in the last 12 months with 40% running out of money for food, clothing, or bills in the last two weeks.

**Table 6:** Employment barriers and finance survey results

Survey Questions	Number	Percent
Problems getting a job due to:		
Own ill health, or disability	30	32
Transport problems or too far to travel	12	13
No jobs at all	7	7
Insufficient education, training or skills	7	7
Too young or too old	6	6
Unable to find suitable childcare	*	*
No jobs in local area or line of work	*	*
Have a criminal record	*	*
Treated badly because you are Aboriginal/Torres Strait Islander/Aboriginal or Torres Strait Islander	*	*
Money available to spend on everyday things is:		
More than enough	7	7
Enough	28	30
Not enough	45	47
Ran out of money for food, clothing or bills in last 2 weeks (yes)	38	40
Times household has experienced problems paying bills in last 12 months		
Never	31	33
Once	*	*
Twice	10	11
3–5	24	25
6–9	*	*
10—19	8	8
20 plus	*	*

*=Cells are < 5 respondents are masked

### The wider environment: broader community, resources and services

The survey explored respondents’ feelings about the community and use of community facilities (see supplementary file 2, Culture and community, Self-determination and Cultural safety sections). Almost all respondents felt their local community was a place where all people are welcomed and valued irrespective of LGBTQ status (87%), religion or spirituality, gender (male respondents 94% and female respondents 91%) and culture (97%). However, only 35% of respondents felt the statement ‘*Aboriginal and Torres Strait Islander culture is valued in Australia’* was ‘often’, ’usually’ or ‘always true’. Approximately a quarter of respondents felt statements that Aboriginal and Torres Strait Islander peoples experience the same rights as other Australians (25%) and do not experience racial discrimination (27%) were ‘often’,’ usually’ or ‘always true’.

A third of respondents reported feeling unfairly treated at least sometimes (33%) with 24% being subjected to racial comments or jokes, 10% being followed by security while shopping and 7% feeling they hadn’t been trusted. Unfair treatment occurred in the public sphere by members of the public (14%) when applying for work (10%), social media (10%) and healthcare settings (7%). Just over half the respondents (53%) agreed with the statement that services were culturally safe was ‘often’, ‘usually’ or ‘always true’. Respondents had difficulty accessing existing services such as social security services (11%), housing services (8%) hospitals (7%) and banks and financial institutions (5%) due to appointment availability (20%), access to services limited by no internet (18%), cost (15%) and poor customer service (13%). Some respondents avoided situations because of past unfair treatment, including going to public places or events (7%), contacting healthcare professionals (6%), contacting services such as police and lawyers (5%) applying for jobs (4%) and sporting or recreational activities (4%).

Respondents felt that a community place to feel culturally safe (32%), cultural events and activities (28%), access to culturally safe employment and training opportunities (26%) and access to services such as an Aboriginal Community Controlled Health Organisation (21%) would be useful in dealing with feelings of being unfairly treated. When asked specifically about a range of facilities and services that were important, 70% of respondents indicated the importance of Aboriginal services provided within mainstream services or Aboriginal controlled service (63%) but indicated less availability with 55% and 56% respectively. A gathering place was important to 53% of respondents.

Education and training support such as subsidies and grants (21%) and homework assistance and tutoring were valued by survey respondents along with opportunities for children to participate in Aboriginal and Torres Strait Islander arts (30%) and language (27%).

### Housing conditions

As shown in Table 7, most respondents indicated that their rental property felt like home (60%) and indicated that their house was at least adequate for their needs (73%). Most households had a range of common appliances with 21% indicating their appliances were not in good working order. Only 40% of respondents had a computer and 46% had internet connection.

**Table 7:** Housing conditions survey results

Questions	Number	Percent
Feels like home		
Yes No	57 18	60 19
Adequacy of housing for needs in general		
Much less than adequate	7	7
Less than adequate	3	3
Adequate	45	47
More than adequate	18	19
Much more than adequate	6	6
Utilities/appliances available		
Stove/oven/cooking facilities	72	76
Fridge	67	71
Heater/heating	65	68
Washing machine	65	68
Laundry tub	58	61
Smoke detectors	64	67
A landline telephone	16	17
A computer	38	41
An internet connection	44	46
A television	62	65
Appliances in working order		
Yes No	55 20	58 21

### Aspirations

When asked their aspirations for the future, most respondents indicated that improving their health and wellbeing (78%) and strengthening relationships with family and friends (73%) was ‘moderately important’, ‘important’ or ‘very important’ (see Table 8).

**Table 8:** Aspirations of Aboriginal peoples living in social housing

Aspirations	Number	Percent
(Response options- not important, slightly important, moderately important, important or very important)	(Responded moderately important, important or very important)	
Improve my health and wellbeing (this may involve a range of activities to improve your health such as giving up smoking or improving the type of food you eat or activities to help you feel more confident and relaxed)	66	78
Strengthen relationships with family and friends (this may involve spending more time with family and friend or improving parenting skills)	62	73
Be involved with culture activities and the local Community (this includes being involved with culturally significant events and local activities run in your community where you meet other people)	57	67
Own assets (this may involve saving to buy a car or things for your home such as furniture or appliances)	55	65
Improve my financial situation (this may involve learning how to manage and save money)	54	64
Develop hobbies, be involved in sports and recreation activities (this might be taking a holiday or joining a sporting club or other activities you enjoy)	52	62
Improve my education and/or employment (e.g. this may involve seeking further skills and training in order to find work in a new area or area of interest)	47	55
Resources to help me get started with a business (start up and ongoing support, equipment)	31	37

## Discussion

This paper presents the results of a survey conducted to understand the SEWB, needs and aspirations of Aboriginal and Torres Strait Islander peoples living in social housing in Victoria, Australia. This study is a first in addressing a gap in understanding the contribution of various domains and determinants to SEWB in this population. An important finding from the study was the identification of a set of strengths by respondents.

### Strengths

The strong sense of identity, connection to family and culture expressed by most survey respondents are key elements of SEWB. These strengths reflect the resilience of Aboriginal and Torres Strait Islander peoples to not only survive changing environments over many thousands of years but also the ongoing impacts of colonialism, genocide, and racism. These strengths can be cultivated by addressing aspirations for enhanced family, community, and cultural connections. Opportunities for greater community and cultural engagement both for children through cultural learning experiences and more broadly through community events and safe places to meet, such as gathering places were identified in the survey. These domains can also inform strengths-based approaches to improving SEWB [15] in line with contemporary approaches in Aboriginal and Torres Strait Islander communities [29, 30].

### Health Burden

Coexisting with the strengths were a set of physical and mental health challenges. The prevalence of health issues was frequently higher among respondents than in the general population of Aboriginal and Torres Strait Islander peoples and the general Australian population. For example, a doctor’s diagnosis of asthma was higher among respondents (36%) than reported by the broader Aboriginal and Torres Strait Islander population in Australia (16%) [31]. Smoking was reported by 41% of survey respondents which is similar to the broader Aboriginal and Torres Strait Islander population aged over 15 in Australia [32] but higher than the 11% rate for all Australians over 18 [33]. Most households included a person with a disability (52%) which is more than the 36% of Australian households [34].

A study of South Australian general population norms indicate a mean self-rated health score of 79 on the EQ-5D-5 L instrument, approximately 9 points higher than our survey group [35]. Norms for Aboriginal and Torres Strait Islander populations and the broader Australian population are not available. The prevalence of back pain among respondents (36%) is higher than in the broader Aboriginal and Torres Strait Islander population aged 2 years and older (12.6%) [31]. These differences reflect the older age of study participants, and the provision of social housing to support people experiencing ill health and disability.

The survey revealed high levels of depression (33%) and anxiety (41%) in Aboriginal peoples living in social housing compared to the broader Aboriginal and Torres Strait Islander population (aged two years and over) with 13% and 17% respectively [32]. The difference again reflects both the age range of participants and their prioritisation for social housing due to complex needs. The rate of reported high or very high levels of psychological distress by survey respondents (28%) is comparable to the 31% of the broader Aboriginal and Torres Strait Islander population not living in remote locations aged over 18 [31] but higher than the general Australian population aged over 16 (15%) experiencing high or very high levels as measured with the K10 [36].

This analysis indicates that at least a quarter of Aboriginal peoples living in social housing experience physical, mental, and emotional conditions at higher rates than the general Australian population and at times higher than the broader Aboriginal and Torres Strait Islander population. This is reflected in at least a third of respondents feeling ‘only a little’ or ‘not at all’ in control of their life, being able to solve problems and make the right choices. Disrupted connections to mind and body can lead to poorer SEWB [11] and hinder engagement in community, cultural activities and broader social, employment and training opportunities. The aspiration most frequently reported by respondents, to improve their health and wellbeing, reflects the desire to address these disrupted connections. Addressing physical and mental health barriers to engagement in their local communities and broader society is not only a need but a priority of Aboriginal peoples living in social housing.

### Social determinants

Additional challenges to SEWB arise from social determinants. Year 12 or certificate III qualification completion rates (45%) among respondents were less than the broader Aboriginal and Torres Strait Islander population aged 20 and over in Australia (57%) [37] reflecting both the younger age range of participants and the complex needs of those living in social housing. Similarly, the rate of paid employment among respondents (18%) was less than that of Aboriginal and Torres Strait Islander peoples in major cities (59%) and in inner regional areas (51%) [38]. High rates of unemployment and inability to work are reflected in more respondents (54%) running out of money for food, clothing or bills than the broader Aboriginal and Torres Strait Islander population (39%) [39]. These disparities reflect barriers to employment including education level, ill health, disability, financial stress, and availability of transport.

Many respondents faced difficulties in accessing services, such as social security and health, and engaging with employment and education due to past negative experiences. The lack of respect for the culture or the rights of Aboriginal and Torres Strait Islander peoples, felt by respondents, along with reports of unfair treatment are significant deterrents to engaging with the broader community and essential services. While persistent racism and inequity are not quickly or easily addressed in Australia, the need for culturally safe and appropriate pathways into health, education, employment, and other key community supports are essential in enabling Aboriginal peoples living in social housing to address their needs and realise their aspirations.

While most survey respondents held aspirations for greater engagement in education, training, and employment, less than half the respondents felt there were opportunities to develop skills in the community or further their education to improve their employment prospects. This may explain why more respondents aspire to improve their financial situation over education or employment, as education and employment may not be seen as easily negotiable pathways to improving finances. Respondents identified the need for further support in the form of subsidies to make training and education more affordable for this population and tutoring to assist school children or coaching for adults.

### Strengths based approaches

Most Aboriginal peoples living in social housing were happy, interested and satisfied with life and have found ways to build identity and important connections to family and community, that can enhance SEWB, despite high rates of health issues, psychosocial and financial distress. Positive psychology has shown that distress and wellbeing can coexist [40] and resilience research has demonstrated people can grow and develop as a result of adversity [41]. Future quantitative research could explore, through regression analysis, the association between strengths and challenges and their respective contribution to wellbeing in this population. This type of research could inform a move beyond deficit based approaches to strength based approaches to promoting wellbeing. Examining various strength-based approaches in Aboriginal and Torres Strait Islander populations, including those living in social housing, will inform whether these approaches are appropriate and effective in improving SEWB. The evaluation of an AHV led coaching strengths based wellbeing intervention, informed by the findings of this survey, will be published once complete and contribute to understanding this approach [15].

### Limitations

The main limitation of the study is the low survey response rate (35%) among AHV tenancy holders. This response rate is comparable to response rates in AHV’s own renter satisfaction survey which returned response rates of between, 17% and 27% in recent years [42–44]. Similar low response rates have been reported in research with social housing tenants internationally [45–47]. The low survey response rate can also be understood in the context of being conducted through repeated and lengthy COVID-19 pandemic lockdowns which added to the stressors for Aboriginal peoples living in social housing.

Survey respondents were significantly older than non-survey participants as the survey was restricted to those 16 years and above. Despite the low response rate and the age difference, the survey sample was found to be largely representative of the AHV tenancy population on several key dimensions related to tenancy stress. Therefore, the study findings are likely to be generalisable to the broader population of people living in AHV social housing. These findings are also likely to be indicative of common issues faced by Aboriginal and Torres Strait Islander peoples in other social housing settings. However, similar research examining the SEWB of Aboriginal and Torres Strait Islander peoples in a range of other community and public housing settings in Australia is needed to understand whether these findings are shared across the broader population.

Self- selection bias may affect the results. Those with more time, resources and experiencing fewer stressors are more likely to respond potentially presenting a more positive picture of social and emotional wellbeing than experienced by non-respondents. The greater proportion of survey respondents from single or single shared household types with fewer caring responsibilities or dependents supports this. The survey’s length and complexity may have also deterred some individuals from participating.

## Conclusions

This study has begun to address a gap in understanding the SEWB, needs and aspirations of Aboriginal peoples living in social housing in Victoria, Australia. Strong connections to identity, family and culture were found to coexist along with disrupted connections to mind, body and community. The strength of these connections, along with challenges in accessing and engaging with employment, education and community resources, can influence SEWB.

The study highlights the need and aspirations for culturally safe and appropriate pathways to services and community resources for Aboriginal and Torres Strait Islander peoples living in social housing to strengthen connections to body, mind, emotions and community. Targeted, co-designed strengths-based programs informed by research could play a role in supporting individuals and communities to enhance connections to key domains of SEWB.

### Electronic supplementary material

Below is the link to the electronic supplementary material.

Supplementary Material 1

Supplementary Material 2

## Data Availability

All data generated or analysed during this study are included in this published article and its supplementary information files.

## Footnotes

1 Note that as no survey respondents identified as Torres Strait Islander that respondents will be referred to as Aboriginal. Where there is reference to the broader Indigenous population the term Aboriginal and Torres Strait Islander is used.

## Abbreviations

AHV: Aboriginal Housing Victoria
ARRQ: Aboriginal Resilience and Recovery Questionnaire
NDIS: National Disability Insurance Scheme
SEWB: Social and Emotional Wellbeing
