# Supplementary material for: Social and emotional wellbeing of Aboriginal and Torres Strait Islander peoples in Aboriginal controlled social housing
Source: BMC Public Health. 2023 Oct 6;23:1935. doi: 10.1186/s12889-023-16817-y (PMC10557265; doi:10.1186/s12889-023-16817-y)
Supplement: Supplementary file 1 — Supplementary Material 1 [file 12889_2023_16817_MOESM1_ESM.docx]

Supplementary File 1

| Table: Housing characteristics among survey and non-survey respondents | | | |  |
| --- | --- | --- | --- | --- |
| **Variables** | **Survey respondents** | **Non-respondents** | **Difference** | **P Value** |
|  | **%(95% CI)** | **%(95% CI)** | **%(95% CI)** |  |
| Gender (Female) | 70.2(60.5,80.0) | 58.9(57.3,60.5) | 11.3(1.5,21.3) | 0.036 |
| Age* (mean, 95% CI) | 45.7(41.9,49.5) | 29.5(28.8,30.1) | 16.2(11.9,20.6) | <0.001 |
| **Rent in arrears** | 23.1(14.4,31.7) | 28.7(27.3,30.2) | -5.7(-14.4,3.1) | 0.238 |
| **Tenancy Related Maintenance Charges (TRMC)** |  |  |  |  |
| Any TRMC | 12.1(5.4,18.8) | 11.9(10.9,13) | 0.2(-6.6,6.9) | 0.964 |
| One TRMC | 9.1(3.1,15.1) | 9.6(8.6,10.5) | -0.5(-6.6,5.6) | 0.875 |
| Two or more TRMC | 7.2(1.7,12.8) | 5.7(4.9,6.5) | 1.5(-4.1,7.1) | 0.559 |
| **Complaints** | 2.2(-0.8,5.2) | 1.9(1.5,2.4) | 0.3(-2.8,3.3) | 0.858 |
| **Household type** |  |  |  |  |
| Single | 34.4(24.6,44.3) | 20.8(19.5,22.1) | 13.6(3.7,23.5) | 0.002 |
| Single shared | 15.6(8.1,23) | 9(8.1,10) | 6.5(-1,14.1) | 0.034 |
| Single with children | 2.2(-0.8,5.3) | 2.6(2.1,3.1) | -0.3(-3.4,2.7) | 0.839 |
| Couple | 40(29.9,50.1) | 59.1(57.5,60.7) | -19.1(-29.3,-8.8) | <0.001 |
| Couple with children | 7.8(2.2,13.3) | 8.5(7.6,9.4) | -0.7(-6.3,4.9) | 0.815 |
| **Housing** |  |  |  |  |
| House | 80(71.5,88.5) | 85.8(84.7,86.9) | -5.8(-14.4,2.8) | 0.132 |
| Unit | 20(11.5,28.5) | 14.2(13.1,15.3) | 5.8(-2.8,14.4) | 0.132 |

*based on age at time 1
