# Supplementary material for: Social and emotional wellbeing of Aboriginal and Torres Strait Islander peoples in Aboriginal controlled social housing
Source: BMC Public Health. 2023 Oct 6;23:1935. doi: 10.1186/s12889-023-16817-y (PMC10557265; doi:10.1186/s12889-023-16817-y)
Supplement: Supplementary file 2 — Supplementary Material 2 [file 12889_2023_16817_MOESM2_ESM.docx]

Supplementary File 2

| **Characteristics** | **Number (N)** | **Percent (%)** |  |  |  |  |
| --- | --- | --- | --- | --- | --- | --- |
| **DEMOGRAPHICS** |  |  |  |  |  |  |
|  |  |  |  |  |  |  |
| **Gender** |  |  |  |  |  |  |
| Female | 67 | 69.8 |  |  |  |  |
| Male | 28 | 30.2 |  |  |  |  |
| **Age** |  |  |  |  |  |  |
| 16-24 | 15 | 15.8 |  |  |  |  |
| 24-34 | 11 | 11.6 |  |  |  |  |
| 35-44 | 21 | 22.1 |  |  |  |  |
| 45-54 | 20 | 21.1 |  |  |  |  |
| 55-64 | 12 | 12.6 |  |  |  |  |
| 65-74 | 11 | 11.6 |  |  |  |  |
| 75 plus | 5 | 5.3 |  |  |  |  |
| **Heritage** |  |  |  |  |  |  |
| Aboriginal | 95 | 100 |  |  |  |  |
| **Relationship status** |  |  |  |  |  |  |
| Single | 56 | 55.8 |  |  |  |  |
| Separated/divorced | 15 | 15.8 |  |  |  |  |
| Married/defacto/living with partner (including same sex partners) | 13 | 13.7 |  |  |  |  |
| In stable relationship but not living together | * | * |  |  |  |  |
| Widowed | * | * |  |  |  |  |
| **Healthcare card or other concession card** |  |  |  |  |  |  |
| Yes | 84 | 88.4 |  |  |  |  |
| No | 10 | 10.5 |  |  |  |  |
| **Member type** |  |  |  |  |  |  |
| Main tenant | 63 | 66.3 |  |  |  |  |
| Joint tenant | 3 | 3.2 |  |  |  |  |
| Household member | 18 | 18.9 |  |  |  |  |
| **HEALTH** |  |  |  |  |  |  |
|  |  |  |  |  |  |  |
| **Advised by Dr of following Health Conditions** | (N=yes) | (% yes) | (N=no) | (% no) |  |  |
| Anxiety or feeling anxious or nervous | 39 | 41.1 | 56 | 58.9 |  |  |
| Back pain or back problems | 34 | 35.8 | 61 | 64.2 |  |  |
| Asthma | 34 | 35.8 | 61 | 64.2 |  |  |
| Depression or feeling depressed | 31 | 32.6 | 64 | 67.4 |  |  |
| Problems with weight | 28 | 29.5 | 67 | 70.5 |  |  |
| High blood pressure | 25 | 26.3 | 70 | 73.7 |  |  |
| Problems with eyes or eyesight | 23 | 24.2 | 72 | 75.8 |  |  |
| Arthritis | 22 | 23.2 | 73 | 76.8 |  |  |
| Problems with mouth, gums or teeth | 18 | 19 | 77 | 81.1 |  |  |
| Trauma | 18 | 19 | 77 | 81.1 |  |  |
| Disability | 16 | 16.8 | 79 | 83.2 |  |  |
| Diabetes | 14 | 14.7 | 81 | 85.3 |  |  |
| Emphysema | 12 | 12.6 | 83 | 87.4 |  |  |
| Cardiovascular Disease | 12 | 12.6 | 83 | 87.4 |  |  |
| Problems with ears or hearing | 9 | 9.5 | 86 | 90.5 |  |  |
| Behavioural or emotional problems | 8 | 8.4 | 87 | 91.6 |  |  |
| Chronic pain that is not back pain | 5 | 5.3 | 90 | 94.7 |  |  |
| Bronchitis | * | * | * | * |  |  |
| Harmful use of/dependence on drugs and/or alcohol | * | * | * | * |  |  |
| Eating disorders | * | * | * | * |  |  |
| Complications with a birth or pregnancy | * | * | * | * |  |  |
| Kidney disease | * | * | * | * |  |  |
| Cancer | * | * | * | * |  |  |
| Stroke | * | * | * | * |  |  |
| Digestive diseases | * | * | * | * |  |  |
| Sexually transmitted infection | * | * | * | * |  |  |
| None of the above | * | * | * | * |  |  |
| Prefer not to answer | * | * | * | * |  |  |
| **Number of Health conditions** |  |  |  |  |  |  |
| No health conditions | 7 | 7.3 |  |  |  |  |
| 1-5 health conditions | 53 | 55.8 |  |  |  |  |
| 6-10 health conditions | 16 | 16.8 |  |  |  |  |
| 11 or more health conditions | 9 | 9.5 |  |  |  |  |
| **Experienced the following health problems in last 6 months** | (N=yes) | (% yes) | (N=no) | (% no) |  |  |
| Pain that is always there or pain that keeps coming back | 36 | 37.9 | 59 | 62.1 |  |  |
| Problems doing things now because of a knock to the head or a stroke | 23 | 24.2 | 72 | 75.8 |  |  |
| Trouble breathing that makes doing things hard | 19 | 20.0 | 76 | 80.0 |  |  |
| Problems with sight or seeing things | 13 | 13.7 | 82 | 86.3 |  |  |
| Fears or any emotional problems for which help is required | 11 | 11.6 | 84 | 88.4 |  |  |
| Any hearing problems | 11 | 11.6 | 84 | 88.4 |  |  |
| Difficulties learning or understanding things | 8 | 8.4 | 87 | 91.6 |  |  |
| Problems concentrating at school or work | 5 | 5.3 | 90 | 94.7 |  |  |
| Blackouts or fits | * | * | * | * |  |  |
| Any problems speaking | * | * | * | * |  |  |
| None of the above | 25 | 27.0 | 70 | 73.7 |  |  |
| **EQ-5D** (Response options = no, slight, moderate, severe, extreme problems) | (N= Slight, moderate or severe or extreme problems) | (% Slight, moderate or severe or extreme problems) | (N=no problems) | (% no problems) |  |  |
| Any problems with usual activities (work study housework, family or leisure) | 44 | 46.3 | 45 | 47.4 |  |  |
| Any problems with mobility | 37 | 38.9 | 52 | 54.7 |  |  |
| Any problems with pain or discomfort | 58 | 61.1 | 31 | 32.6 |  |  |
| Any problems with depression or anxiety | 50 | 52.6 | 39 | 41.1 |  |  |
| Any problems with personal care (washing and dressing) | 13 | 13.7 | 76 | 80.0 |  |  |
| EQOL - how good or bad is your health today (out of 100) | - | 70 (median) |  |  |  |  |
| **Disability** | (N=yes) | (% yes) | (N=no) | (% no) |  |  |
| Do you or anybody in your household suffer from a disability? | 49 | 51.6 | 30 | 31.6 |  |  |
| Do you or anyone in the household know what the National Disability Insurance Scheme (NDIS) is? | 43 | 45.2 | 28 | 29.5 |  |  |
| Do you know if anyone in your household is currently receiving supports funded by the NDIS | 21 | 22.1 | 55 | 57.9 |  |  |
| Is there anyone in this household that could be accessing NDIS but isn’t? | 20 | 21.1 | 52 | 54.7 |  |  |
| **Smoking** |  |  |  |  |  |  |
| Do you smoke cigarettes or any other tobacco products? |  |  |  |  |  |  |
| Yes | 39 | 41.1 |  |  |  |  |
| No, I have given up smoking | 26 | 27.4 |  |  |  |  |
| No, I have never smoked | 17 | 17.9 |  |  |  |  |
| If “Yes”, are you interested in getting help to quit? | 12 | 30.8 |  |  |  |  |
| **WELLBEING** |  |  |  |  |  |  |
|  |  |  |  |  |  |  |
| **Kessler** |  |  |  |  |  |  |
| Kessler 5 score (range 0-25) |  | 10 (median) |  |  |  |  |
| % Experiencing high/very high psychological distress | 27 | 28.4 |  |  |  |  |
| (score 12-25) ref ABS |  |  |  |  |  |  |
| % Experiencing moderate/low psychological distress (1-11) | 62 | 65.3 |  |  |  |  |
| (score 5-11) ref ABS |  |  |  |  |  |  |
| **Mental Health Continuum** (Response options: Never/once or twice/About once a week/2-3 times a week/almost everyday/everyday) | (N= either about 2-3 times a week/almost everyday/everyday) | (% either about 2-3 times a week/almost everyday/everyday) | (N=about once a week or once or twice) | (% about once a week or once or twice) | (N= never) | (% never) |
| **Emotional wellbeing-** During the past month, how often did you feel |  |  |  |  |  |  |
| Happy | 69 | 72.6 | 15 | 15.8 | 5 | 16.6 |
| Interested in life | 67 | 70.5 | 16 | 16.8 | 6 | 17.7 |
| Satisfied with life | 60 | 63.2 | 21 | 22.1 | 8 | 23.3 |
| **Psychological wellbeing** |  |  |  |  |  |  |
| During the past month, how often did you feel  That you liked most parts of your personality | 66 | 69.5 | 18 | 18.9 | 5 | 19.9 |
| Good at managing the responsibilities of your daily life | 74 | 77.9 | 12 | 12.6 | * | * |
| That you had warm and trusting relationships with others | 65 | 68.4 | 15 | 15.8 | 9 | 16.6 |
| That you had experiences that challenged you to grow and become a better person | 64 | 67.4 | 15 | 15.8 | 10 | 16.6 |
| Confident to think or express your own ideas and opinions | 68 | 71.6 | 15 | 15.8 | 6 | 16.6 |
| That your life has a sense of direction or meaning to it | 64 | 67.4 | 16 | 16.8 | 9 | 17.7 |
| emotional well-being 3 items (range 0-15) |  | 11 (median) |  |  |  |  |
| psychological well-being – 6 items (range 0-30) |  | 23 (median) |  |  |  |  |
| MHC total (range 0-45) |  | 32 (median) |  |  |  |  |
| **ARRQ** (Response options: not at all/a little/somewhat/a fair bit/a lot) | (N= a fair bit/a lot) | (% a fair bit/a lot) | (N=somewhat) | (% somewhat) | (N=a little/not at all) | (% a little/not at all) |
| I am able to maintain my Aboriginal or Torres Strait Islander identity, values and beliefs | 63 | 66.3 | 10 | 10.5 | 16 | 16.8 |
| I feel supported by my friends/mob | 57 | 60 | 15 | 15.8 | 17 | 17.9 |
| In my community I have opportunities to develop skills (e.g., job skills or skills to care for others) | 43 | 45.3 | 21 | 22.1 | 25 | 26.3 |
| I can trust myself to make the right choice | 61 | 64.2 | 14 | 14.7 | 14 | 14.7 |
| Being Aboriginal or Torres Strait Islander is an important part of who I am | 73 | 76.8 | 8 | 8.4 | 8 | 8.4 |
| I have family that love me even when I muck up | 75 | 78.9 | 2 | 2.1 | 12 | 12.6 |
| Spirituality is a source of strength for me | 63 | 66.3 | 10 | 10.5 | 16 | 16.8 |
| I am able to deal with most problems that occur in my life | 64 | 67.4 | 18 | 18.9 | 7 | 7.4 |
| I have opportunities to work in my life, keep busy and stay involved | 50 | 52.6 | 16 | 16.8 | 23 | 24.2 |
| There are people in my life that I have close, secure relationships with | 61 | 64.2 | 12 | 12.6 | 16 | 16.8 |
| I am proud to be Aboriginal or Torres Strait Islander | 76 | 80 | 4 | 4.2 | 9 | 9.5 |
| When changes occur in my life I can usually find ways to adapt | 59 | 62.1 | 19 | 20.0 | 11 | 11.6 |
| In my community I have opportunities to further my education | 46 | 48.4 | 16 | 16.8 | 17 | 17.9 |
| I can turn to my partner or someone close to me for support and understanding | 55 | 57.9 | 18 | 18.9 | 16 | 16.8 |
| I am able to have a laugh even when things are difficult | 57 | 60 | 17 | 17.9 | 15 | 15.8 |
| I take positive action to try and solve problems | 59 | 62.1 | 17 | 17.9 | 13 | 13.7 |
| I participate in cultural practices that give me peace (such as going out bush, ceremony, community cultural events) | 39 | 41.1 | 21 | 22.1 | 29 | 30.5 |
| I feel safe when I am with my partner or those closest to me | 69 | 72.6 | 6 | 6.3 | 14 | 14.7 |
| I find it easy to get along well with people | 59 | 62.1 | 14 | 14.7 | 16 | 16.8 |
| Overall, I feel like I have control over my life | 54 | 56.8 | 22 | 23.2 | 13 | 13.7 |
| I have the skills to be confident in both Indigenous and non-Indigenous communities | 56 | 58.9 | 18 | 18.9 | 15 | 15.8 |
| I feel safe when I am with my family | 71 | 74.7 | 7 | 7.4 | 11 | 11.6 |
| I feel confident in socialising with others around me | 51 | 53.7 | 21 | 22.1 | 17 | 17.9 |
| I feel content with my life | 61 | 64.2 | 14 | 14.7 | 14 | 14.7 |
| I speak an Aboriginal or Torres Strait Islander language(s) | 17 | 17.9 | 10 | 10.5 | 62 | 65.3 |
| **FAMILY** |  |  |  |  |  |  |
|  |  |  |  |  |  |  |
| **How many dependents under the age of 18 do you support** |  |  |  |  |  |  |
| 0 | 57 | 60.0 |  |  |  |  |
| 1 | 13 | 13.7 |  |  |  |  |
| 2 | 8 | 8.4 |  |  |  |  |
| 3 | 9 | 9.5 |  |  |  |  |
| 4 | * | * |  |  |  |  |
| 5 | * | * |  |  |  |  |
| **Ages of dependents** |  |  |  |  |  |  |
| 0-4 | 14 |  |  |  |  |  |
| 5-9 | 20 |  |  |  |  |  |
| 10-14 | 19 |  |  |  |  |  |
| 15-17 | 15 |  |  |  |  |  |
| **Relationship of dependents** |  |  |  |  |  |  |
| Child | 47 | 90.4 |  |  |  |  |
| Grandchild | * | * |  |  |  |  |
| Sister/brother | * | * |  |  |  |  |
| cousin | * | * |  |  |  |  |
| Step child | * | * |  |  |  |  |
| **Strength based parenting**  (Response options strongly agree/agree/neither agree nor disagree/disagree/strongly disagree) | (N=Agree or strongly agree) | (% Agree or strongly agree) | (N=Neither agree nor disagree/ disagree/r strongly disagree) | (% Neither agree nor disagree/ disagree/ strongly disagree) |  |  |
| I know the things my kids are good at doing | 29 | 76.3 | 9 | 9.5 |  |  |
| I am aware of the strengths my kids have | 31 | 81.6 | 7 | 7.4 |  |  |
| I show my kids how to use their strengths in different situations | 30 | 78.9 | 8 | 8.4 |  |  |
| I give my kids lots of opportunities to use their strengths | 30 | 78.9 | 8 | 8.4 |  |  |
| **Education, Employment & Finances** |  |  |  |  |  |  |
|  |  |  |  |  |  |  |
| **Highest completed education level** |  |  |  |  |  |  |
| Never went to school | * | * |  |  |  |  |
| Attended primary school but did not finish | * | * |  |  |  |  |
| finished primary school | 5 | 5.3 |  |  |  |  |
| Year 7 | 7 | 7.4 |  |  |  |  |
| Year 8 | * | * |  |  |  |  |
| Year 9 | * | * |  |  |  |  |
| Year 10 | 13 | 13.7 |  |  |  |  |
| Year 11 | 11 | 11.6 |  |  |  |  |
| Year 12 | 10 | 10.5 |  |  |  |  |
| Certificate I/II | * | * |  |  |  |  |
| Certificate III/IV (including trade certificate) | 16 | 16.8 |  |  |  |  |
| Advanced diploma | 8 | 8.4 |  |  |  |  |
| Bachelor degree (with or without honours) | 5 | 5.3 |  |  |  |  |
| Graduate diploma/graduate certificate | * | * |  |  |  |  |
| Post-graduate degree | * | * |  |  |  |  |
| **Current Employment status** |  |  |  |  |  |  |
| Unable to work | 23 | 24.2 |  |  |  |  |
| In paid employment(This can include full-time, part-time, casual and cash in hand work) | 17 | 17.9 |  |  |  |  |
| Out of work | 16 | 16.8 |  |  |  |  |
| Retired | 14 | 14.7 |  |  |  |  |
| A homemaker | 11 | 11.6 |  |  |  |  |
| In secondary school | * | * |  |  |  |  |
| Studying | * | * |  |  |  |  |
| **Currently looking for work** |  |  |  |  |  |  |
| Yes | 17 | 17.9 |  |  |  |  |
| No | 71 | 74.7 |  |  |  |  |
| **Problems getting a job** |  |  |  |  |  |  |
| Own ill health, or disability | 30 | 31.6 |  |  |  |  |
| Transport problems or too far to travel | 12 | 12.6 |  |  |  |  |
| No jobs at all | 7 | 7.4 |  |  |  |  |
| Insufficient education, training or skills | 7 | 7.4 |  |  |  |  |
| Too young or too old | 6 | 6.3 |  |  |  |  |
| Unable to find suitable child care | * | * |  |  |  |  |
| No jobs in local area or line of work | * | * |  |  |  |  |
| Have a criminal record | * | * |  |  |  |  |
| Treated badly because you are Aboriginal/Torres Strait Islander/Aboriginal or Torres Strait Islander | * | * |  |  |  |  |
| Don't have a drivers license | * | * |  |  |  |  |
| No difficulties/problems getting a job | * | * |  |  |  |  |
| **Money available to spend on everyday things** |  |  |  |  |  |  |
| More than enough | 7 | 7.4 |  |  |  |  |
| Enough | 28 | 29.5 |  |  |  |  |
| Not enough | 45 | 47.4 |  |  |  |  |
| **Times household has experienced problems paying bills in last 12 months** |  |  |  |  |  |  |
| Never | 31 | 32.6 |  |  |  |  |
| once | * | * |  |  |  |  |
| twice | 10 | 10.5 |  |  |  |  |
| 3-5 | 24 | 25.3 |  |  |  |  |
| 6-9 | * | * |  |  |  |  |
| 10--19 | 8 | 8.4 |  |  |  |  |
| 20 plus | * | * |  |  |  |  |
| **Ran out of money for food, clothing or bills in last 12 months** |  |  |  |  |  |  |
| Yes | 51 | 53.7 |  |  |  |  |
| No | 39 | 41.1 |  |  |  |  |
| **Ran out of money for food, clothing or bills in last 2 weeks** |  |  |  |  |  |  |
| Yes | 38 | 40 |  |  |  |  |
| No | 53 | 55.8 |  |  |  |  |
| **Personal Fortnightly income from all sources** |  |  |  |  |  |  |
| None | * | * |  |  |  |  |
| $1 - $99 | * | * |  |  |  |  |
| $100 - $199 | * | * |  |  |  |  |
| $200 - $499 | 23 | 24.2 |  |  |  |  |
| $500 - $999 | 33 | 34.8 |  |  |  |  |
| $1000 - $1499 | 9 | 9.5 |  |  |  |  |
| More than $1500 | 10 | 10.5 |  |  |  |  |
| **This income received every fortnight** |  |  |  |  |  |  |
| Yes | 74 | 77.1 |  |  |  |  |
| No | 10 | 10.5 |  |  |  |  |
| **Family Violence** |  |  |  |  |  |  |
|  |  |  |  |  |  |  |
| Felt unsafe or afraid in last 12 months | 10 | 10.5 |  |  |  |  |
| Controlled or been put down in last 12 months | 7 | 7.4 |  |  |  |  |
| Been threatened with physical harm in last 12 months | 8 | 8.4 |  |  |  |  |
| Been subject to physical harm in last 12 months | * | * |  |  |  |  |
| **Self-determination (Response options not true at all/sometimes /often/usually/always true)** | (N= often, usually or always true) | (% often, usually or always true) | (N=sometimes) | (% sometimes) | (N=not at all true) | (% not at all true) |
| Aboriginal and Torres Strait Islander people experience the same rights as other Australians | 24 | 25.3 | 39 | 41.1 | 26 | 27.4 |
| Aboriginal and Torres Strait Islander culture is valued in Australia | 33 | 34.7 | 36 | 37.9 | 20 | 21.1 |
| Service providers recognise and promote the right of Aboriginal Peoples to people to make their own choices and decisions and to manage their own affairs | 39 | 41.1 | 37 | 38.9 | 13 | 13.7 |
| Service providers respect and support Aboriginal people to make their own choices and decisions when using services | 44 | 46.3 | 34 | 35.8 | 11 | 11.6 |
| Decision about services for the community are decided by partnerships led by Aboriginal people/organisations | 42 | 44.2 | 37 | 38.9 | 10 | 10.5 |
| Power to make decisions for the community is being shifted to Aboriginal and Torres Strait Islander people/organisations | 45 | 47.4 | 32 | 33.7 | 12 | 12.6 |
| Aboriginal and Torres Strait Islander people/organisations lead the design of service delivery for their community | 51 | 53.7 | 27 | 28.4 | 11 | 11.6 |
| Services provided to Aboriginal and Torres Strait Islander people are culturally safe | 50 | 52.6 | 35 | 36.8 | 4 | 4.2 |
| Funding to Aboriginal people/organisations is directed to providing services and supports that assist in achieving community aspirations | 47 | 49.5 | 32 | 33.7 | 10 | 10.5 |
| Aboriginal and Torres Strait Islander people do no experience racial discrimination | 26 | 27.4 | 25 | 26.3 | 38 | 40.0 |
| Opportunities exist to question and review whether services delivered by organisations are improving outcomes for Aboriginal and Torres Strait Islander people | 40 | 42.1 | 40 | 42.1 | 9 | 9.5 |
| **Culture and Community** |  |  |  |  |  |  |
| **Knowledge of traditional country or homeland** |  |  |  |  |  |  |
| Yes | 82 | 86.3 |  |  |  |  |
| No | * | * |  |  |  |  |
| No but would like to know | * | * |  |  |  |  |
| Currently exploring | * | * |  |  |  |  |
| **Knowledge of mob/mobs** |  |  |  |  |  |  |
| Yes | 76 | 80 |  |  |  |  |
| No | 9 | 9.5 |  |  |  |  |
| No, but would like to know | 6 | 6.3 |  |  |  |  |
| **Involvement in Ceremony in last 12 months** |  |  |  |  |  |  |
| NAIDOC week activities | 31 | 32.3 |  |  |  |  |
| Funerals/sorry business | 24 | 25 |  |  |  |  |
| Been involved with any Aboriginal or Torres Strait Islander Organisations | 19 | 19.8 |  |  |  |  |
| Celebration/ceremonies | 14 | 14.7 |  |  |  |  |
| Festivals or carnivals involving arts, craft, music or dance | 11 | 11.5 |  |  |  |  |
| Caring for Country | 8 | 8.3 |  |  |  |  |
| Sports Carnivals | 8 | 8.3 |  |  |  |  |
| Fishing | 8 | 8.3 |  |  |  |  |
| Gathering plants/berries | 6 | 6.3 |  |  |  |  |
| Hunting | * | * |  |  |  |  |
| None of the above | * | * |  |  |  |  |
| **Participate in cultural activities as often as want** |  |  |  |  |  |  |
| Yes | 55 | 57.9 |  |  |  |  |
| No | 30 | 31.6 |  |  |  |  |
| **Problems participating in ceremony (N=30)** |  |  |  |  |  |  |
| Can't afford to | 10 | 33.3 |  |  |  |  |
| Too far away | 8 | 26.7 |  |  |  |  |
| Access to the knowledge holders | * | * |  |  |  |  |
| Caring commitments | * | * |  |  |  |  |
| Work commitments | * | * |  |  |  |  |
| Transport problems | 0 | 0 |  |  |  |  |
| School/study commitments | 0 | 0 |  |  |  |  |
| Finding out about cultural activities/ceremonies | 0 | 0 |  |  |  |  |
| **Local Community attitudes** (Response options: not true at all/not usually true/sometimes true/often true/almost always true) | (N= sometimes, often or almost always true) | (% sometimes, often or almost always true) | (N=not true or not usually true) | (% not true or not usually true) |  |  |
| A place where all cultures are welcome and valued | 92 | 96.8 | 2 | 2.1 |  |  |
| A place where Lesbian Gay Bisexual Transgender Queer (LGBTQ) peoples and people from related communities are welcome and valued | 83 | 87.4 | 10 | 10.5 |  |  |
| A placed where religion and spirituality are welcome and valued | 83 | 87.4 | 6 | 6.3 |  |  |
| A place where environmental decision making is valued and respected | 83 | 87.4 | 9 | 9.5 |  |  |
| Women are valued and respected | 86 | 90.5 | 6 | 6.3 |  |  |
| Men are valued and respected | 89 | 93.7 | 2 | 2.1 |  |  |
| Community have control over decision making | 72 | 75.8 | 19 | 20.0 |  |  |
| **Service Use** |  |  |  |  |  |  |
| **Facilities Importance** (Response options: very important/moderately important/not important) | (N=very important) | (% very important) | (N= moderately important/not important) | (% moderately important/not important) |  |  |
| Supermarket | 68 | 71.6 | 7 | 7.4 |  |  |
| Children's playgrounds | 54 | 56.8 | 21 | 22.1 |  |  |
| Gathering place | 50 | 52.6 | 23 | 24.2 |  |  |
| Community hall or centre | 47 | 49.5 | 25 | 26.3 |  |  |
| Outdoor playing fields | 53 | 55.8 | 22 | 23.2 |  |  |
| Taxi Service | 57 | 60 | 14 | 14.7 |  |  |
| Swimming pool | 49 | 51.6 | 25 | 26.3 |  |  |
| Library | 53 | 55.8 | 21 | 22.1 |  |  |
| Child care (prep and/or kindy) | 59 | 62.1 | 13 | 13.7 |  |  |
| Sports clubs (football, netball) | 56 | 58.9 | 19 | 20.0 |  |  |
| Primary school | 60 | 63.2 | 14 | 14.7 |  |  |
| Secondary school | 56 | 58.9 | 14 | 14.7 |  |  |
| Pubs/Restaurant | 43 | 45.3 | 32 | 33.7 |  |  |
| Cinema | 40 | 42.1 | 34 | 35.8 |  |  |
| Homework club | 45 | 47.4 | 26 | 27.4 |  |  |
| Parents rooms | 50 | 52.6 | 21 | 22.1 |  |  |
| Disability access | 55 | 57.9 | 17 | 17.9 |  |  |
| Youth groups/hubs | 53 | 55.8 | 19 | 20.0 |  |  |
| Opportunity to volunteer | 50 | 52.6 | 20 | 21.1 |  |  |
| **Facilities Use** | (N=yes) | (% yes) | (N=no) | (%=no) |  |  |
| Children's playgrounds | 36 | 37.9 | 50 | 52.6 |  |  |
| Gathering place | 30 | 31.6 | 55 | 57.9 |  |  |
| Community hall or centre | 28 | 29.5 | 55 | 57.9 |  |  |
| Outdoor playing fields | 41 | 43.2 | 44 | 46.3 |  |  |
| Supermarket | 81 | 85.3 | 5 | 5.3 |  |  |
| Taxi Service | 46 | 48.4 | 39 | 41.1 |  |  |
| Swimming pool | 43 | 45.3 | 41 | 43.2 |  |  |
| Library | 37 | 38.9 | 47 | 49.5 |  |  |
| Child care (prep and/or kindy) | 23 | 24.2 | 59 | 62.1 |  |  |
| Sports clubs (football, netball) | 37 | 38.9 | 51 | 53.7 |  |  |
| Primary school | 31 | 32.6 | 54 | 56.8 |  |  |
| Secondary school | 29 | 30.5 | 55 | 57.9 |  |  |
| Pubs/Restaurant | 58 | 61.1 | 30 | 31.6 |  |  |
| Cinema | 50 | 52.6 | 35 | 36.8 |  |  |
| Homework club | 12 | 12.6 | 70 | 73.7 |  |  |
| Parents rooms | 23 | 24.2 | 59 | 62.1 |  |  |
| Disability access | 21 | 22.1 | 63 | 66.3 |  |  |
| Youth groups/hubs | 20 | 21.1 | 64 | 67.4 |  |  |
| Opportunity to volunteer | 26 | 27.4 | 56 | 58.9 |  |  |
| **Facilities Availability** | (N=yes) | (% yes) | (N=no) | (%=no) |  |  |
| Children's playgrounds | 61 | 64.2 | 1 | 1.1 |  |  |
| Gathering place | 35 | 36.8 | 13 | 13.7 |  |  |
| Community hall or centre | 41 | 43.2 | 11 | 11.6 |  |  |
| Outdoor playing fields | 60 | 63.2 | 4 | 4.2 |  |  |
| Supermarket | 73 | 76.8 | 2 | 2.1 |  |  |
| Taxi Service | 60 | 63.2 | 5 | 5.3 |  |  |
| Swimming pool | 62 | 65.3 | 7 | 7.4 |  |  |
| Library | 63 | 66.3 | 5 | 5.3 |  |  |
| Child care (prep and/or kindy) | 52 | 54.7 | 9 | 9.5 |  |  |
| Sports clubs (football, netball) | 58 | 61.1 | 7 | 7.4 |  |  |
| Primary school | 61 | 64.2 | 0 | 0.0 |  |  |
| Secondary school | 59 | 62.1 | 6 | 6.3 |  |  |
| Pubs/Restaurant | 65 | 68.4 | 6 | 6.3 |  |  |
| Cinema | 57 | 60 | 9 | 9.5 |  |  |
| Homework club | 35 | 36.8 | 12 | 12.6 |  |  |
| Parents rooms | 35 | 36.8 | 12 | 12.6 |  |  |
| Disability access | 51 | 53.7 | 6 | 6.3 |  |  |
| Youth groups/hubs | 48 | 50.5 | 8 | 8.4 |  |  |
| Opportunity to volunteer | 50 | 52.6 | 4 | 4.2 |  |  |
| **Service Importance** (Response options: very important/moderately important/not important) | (N=very important) | (% very important) | (N= moderately important/not important) | (% moderately important/not important) |  |  |
| Aboriginal services within a mainstream services | 66 | 69.5 | 6 | 73.2 |  |  |
| Aboriginal controlled service | 60 | 63.2 | 12 | 66.5 |  |  |
| Dentist | 65 | 68.4 | 5 | 72.0 |  |  |
| Medical (GP) | 63 | 66.3 | 6 | 69.8 |  |  |
| Allied health | 54 | 56.8 | 11 | 59.8 |  |  |
| Aged care | 57 | 60 | 13 | 63.2 |  |  |
| Family and relationship support services | 57 | 60 | 10 | 63.2 |  |  |
| Alternative health provider | 55 | 57.9 | 12 | 60.9 |  |  |
| **Service Use** | (N=yes) | (% yes) | (N=no) | (%=no) |  |  |
| Aboriginal services within a mainstream services | 59 | 62.1 | 29 | 30.5 |  |  |
| Aboriginal controlled service | 63 | 66.3 | 21 | 22.1 |  |  |
| Dentist | 67 | 70.5 | 19 | 20.0 |  |  |
| Medical (GP) | 72 | 75.8 | 10 | 10.5 |  |  |
| Allied health | 43 | 45.3 | 38 | 40.0 |  |  |
| Aged care | 25 | 26.3 | 56 | 58.9 |  |  |
| Family and relationship support services | 27 | 28.4 | 57 | 60.0 |  |  |
| Alternative health provider | 39 | 41.1 | 45 | 47.4 |  |  |
| **Service Availability** | (N=yes) | (% yes) | (N=no) | (%=no) |  |  |
| Aboriginal services within a mainstream services | 52 | 54.7 | 6 | 6.3 |  |  |
| Aboriginal controlled service | 53 | 55.8 | 11 | 11.6 |  |  |
| Dentist | 62 | 65.3 | 6 | 6.3 |  |  |
| Medical (GP) | 66 | 69.5 | 3 | 3.2 |  |  |
| Allied health | 51 | 53.7 | 6 | 6.3 |  |  |
| Aged care | 52 | 54.7 | 8 | 8.4 |  |  |
| Family and relationship support services | 48 | 50.5 | 9 | 9.5 |  |  |
| Alternative health provider | 47 | 49.5 | 7 | 7.4 |  |  |
| **Services that would be helpful in the future** |  |  |  |  |  |  |
| After school activities in the local community | 28 | 29.5 |  |  |  |  |
| A place for my children to learn, participate in and perform Aboriginal and/or Torres Strait Islander art, craft, music, dance or theatre | 28 | 29.5 |  |  |  |  |
| Opportunities for my children to learn an Aboriginal and/or Torres Strait Islander language at school or in the local community | 26 | 27.4 |  |  |  |  |
| A place for my children to hear stories in Aboriginal and/or Torres Strait Islander languages | 24 | 25.3 |  |  |  |  |
| Access to good primary and high schools | 21 | 22.1 |  |  |  |  |
| Access to childcare and schools that acknowledge Aboriginal and/or Torres Strait Islander culture in the curriculum | 19 | 20 |  |  |  |  |
| Support at school from teachers | 19 | 20 |  |  |  |  |
| Cooking classes | 18 | 18.9 |  |  |  |  |
| Support for me to help my children with their school work | 17 | 17.9 |  |  |  |  |
| Access to a community garden | 16 | 16.8 |  |  |  |  |
| Access to high quality early learning services in the local area | 15 | 15.8 |  |  |  |  |
| Access to role models in the community | 14 | 14.7 |  |  |  |  |
| Information about healthy food | 12 | 12.6 |  |  |  |  |
| **Children attend the following** |  |  |  |  |  |  |
| Formal child care or early learning centre | 10 | 10.5 |  |  |  |  |
| Family Daycare | * | * |  |  |  |  |
| Kindergarten (pre-school) | * | * |  |  |  |  |
| Primary school (Prep to Grade 6) | 21 | 22.1 |  |  |  |  |
| High school (Years 7 to 12) | 14 | 14.7 |  |  |  |  |
| Children do not attend any of the above | 6 | 6.3 |  |  |  |  |
| **If not attend preschool or school why not** |  |  |  |  |  |  |
| Too old or too young to attend | 14 | 14.7 |  |  |  |  |
| Not culturally safe | 0 | 0 |  |  |  |  |
| Too far to travel | 0 | 0 |  |  |  |  |
| Too expensive | 4 | 4.2 |  |  |  |  |
| My child/ren refuses to go to childcare, kindergarten, school | * | * |  |  |  |  |
| My child needs support to attend | * | * |  |  |  |  |
| **Interest in family reconnection services** |  |  |  |  |  |  |
| Family Tracing | 28 | 29.5 |  |  |  |  |
| Reunion Services | 10 | 10.5 |  |  |  |  |
| Counselling | 10 | 10.5 |  |  |  |  |
| **Health and wellbeing supports required now or in future** |  |  |  |  |  |  |
| A health check | 35 | 36.8 |  |  |  |  |
| An eye check | 27 | 28.4 |  |  |  |  |
| A hearing check | 21 | 22.1 |  |  |  |  |
| Community exercise programs | 19 | 20 |  |  |  |  |
| Access to a psychologist or counselling service | 18 | 18.9 |  |  |  |  |
| Access to free or low cost exercise programs | 17 | 17.9 |  |  |  |  |
| Information about healthy food | 15 | 15.8 |  |  |  |  |
| Information about my mental health and wellbeing | 15 | 15.8 |  |  |  |  |
| Help to quit smoking | 13 | 13.7 |  |  |  |  |
| Advice and help with drug and alcohol addiction | 5 | 5.3 |  |  |  |  |
| Help at school to learn and/or concentrate | 5 | 5.3 |  |  |  |  |
| Help to quit or cut down using yarndi | * | * |  |  |  |  |
| Assistance at home | * | * |  |  |  |  |
| Access to drug and alcohol support | * | * |  |  |  |  |
| Help or support to connect with community | * | * |  |  |  |  |
| **Education and training supports required now or in future** |  |  |  |  |  |  |
| Subsidies or grants to help affordability | 20 | 21.1 |  |  |  |  |
| Career guidance | 9 | 9.5 |  |  |  |  |
| Assistance for students with disabilities | 9 | 9.5 |  |  |  |  |
| A local community homework club | 9 | 9.5 |  |  |  |  |
| Literacy and/or numeracy support | 8 | 8.4 |  |  |  |  |
| Individual tutoring | 8 | 8.4 |  |  |  |  |
| Provision of coaches/mentors | 7 | 7.4 |  |  |  |  |
| Support from the local council | 6 | 6.3 |  |  |  |  |
| Suitable, reliable and accessible transport | 6 | 6.3 |  |  |  |  |
| Help with childcare to attend school or study | 6 | 6.3 |  |  |  |  |
| Access to apprenticeships | 5 | 5.3 |  |  |  |  |
| A quiet place at home to study or do homework | * | * |  |  |  |  |
| Schools suitable for culture and beliefs | * | * |  |  |  |  |
| Better access to local high schools | * | * |  |  |  |  |
| Resources to help you get started with a business (startup finances and information, ongoing support and equipment) | 0 | 0 |  |  |  |  |
| **Difficulty accessing any of these services** |  |  |  |  |  |  |
| Centrelink | 10 | 10.5 |  |  |  |  |
| Housing services | 8 | 8.4 |  |  |  |  |
| Hospitals | 7 | 7.4 |  |  |  |  |
| Banks, credit unions or other financial institutions | 5 | 5.3 |  |  |  |  |
| Aboriginal or Torres Strait Islander health workers | 5 | 5.3 |  |  |  |  |
| Phone Provider | * | * |  |  |  |  |
| Alcohol and drug services | * | * |  |  |  |  |
| Disability services | * | * |  |  |  |  |
| Legal services | * | * |  |  |  |  |
| Mental health services such as counselling | * | * |  |  |  |  |
| Medicare | * | * |  |  |  |  |
| Power, water or gas providers | * | * |  |  |  |  |
| Employment services family assistance office | 0 | 0 |  |  |  |  |
| Doctors | 0 | 0 |  |  |  |  |
| Dentists | 0 | 0 |  |  |  |  |
| internet Provider | 0 | 0 |  |  |  |  |
| Motor vehicle registry | 0 | 0 |  |  |  |  |
| No problems accessing service providers | 55 | 57.9 |  |  |  |  |
| Have not tried to access any services providers | * | * |  |  |  |  |
| **Problem or difficulty accessing services (N=40)** |  |  |  |  |  |  |
| Waiting too long/appointment not available at time required | 8 | 20 |  |  |  |  |
| Don't have internet to access online services | 7 | 17.5 |  |  |  |  |
| Cost of service | 6 | 15 |  |  |  |  |
| Poor customer service | 5 | 12.5 |  |  |  |  |
| Cannot trust them | * | * |  |  |  |  |
| Disability restricts access to service | * | * |  |  |  |  |
| Language difficulties | * | * |  |  |  |  |
| No service in your area | * | * |  |  |  |  |
| Transport/distance | * | * |  |  |  |  |
| Treated badly because I’m Aboriginal/Torres Strait Islander/Aboriginal or Torres Strait Islander | * | * |  |  |  |  |
| Inadequate services in your area | * | * |  |  |  |  |
| Service not culturally appropriate | * | * |  |  |  |  |
| **HOUSING** |  |  |  |  |  |  |
| **Are you the person named on the tenancy agreement?** | 69 | 72.6 |  |  |  |  |
| **How long have you lived in this home?** |  |  |  |  |  |  |
| 0-6 months | * | * |  |  |  |  |
| 7-12 months | * | * |  |  |  |  |
| 1-2 years | 11 | 11.6 |  |  |  |  |
| 3-5 years | 12 | 12.6 |  |  |  |  |
| 6-10 years | 23 | 24.2 |  |  |  |  |
| 11 years or more | 32 | 33.7 |  |  |  |  |
| **Number of people over the age of 18 who currently live at your home** |  |  |  |  |  |  |
| 1 | 53 | 55.8 |  |  |  |  |
| 2 | 19 | 20 |  |  |  |  |
| 3 | 10 | 10.5 |  |  |  |  |
| 4 | * | * |  |  |  |  |
| 5 | * | * |  |  |  |  |
| **Age of occupants over the age of 18** |  |  |  |  |  |  |
| 18-24 | 27 | 28.4 |  |  |  |  |
| 25-64 | 47 | 49.5 |  |  |  |  |
| 65 plus | 13 | 13.7 |  |  |  |  |
| **Gender of occupants over the age of 18** |  |  |  |  |  |  |
| Male | 36 | 37.9 |  |  |  |  |
| Female | 56 | 58.9 |  |  |  |  |
| **Employment status of occupants over the age of 18** |  |  |  |  |  |  |
| Employed | 20 | 21.1 |  |  |  |  |
| Not employed | 60 | 63.2 |  |  |  |  |
| **Financial pressure because of the tenancy** |  |  |  |  |  |  |
| Yes | 5 | 5.3 |  |  |  |  |
| No | 62 | 65.3 |  |  |  |  |
| **Willingness to contact AHV about financial difficulties** |  |  |  |  |  |  |
| Yes | 43 | 45.3 |  |  |  |  |
| No | 21 | 22.1 |  |  |  |  |
| **Previous dwelling** |  |  |  |  |  |  |
| Home Owner | * | * |  |  |  |  |
| Another social housing dwelling | 19 | 20 |  |  |  |  |
| Private rental | 18 | 18.9 |  |  |  |  |
| Living with someone else and you had to move out | 9 | 9.5 |  |  |  |  |
| Share house | 8 | 8.4 |  |  |  |  |
| Institution | 0 | 0 |  |  |  |  |
| Supported accommodation | 0 | 0 |  |  |  |  |
| rehabilitation | 0 | 0 |  |  |  |  |
| Moved around, no fixed address | * | * |  |  |  |  |
| Homeless | 5 | 5.3 |  |  |  |  |
| Prefer not to answer | 15 | 15.8 |  |  |  |  |
| Share accommodation | * | * |  |  |  |  |
| **Length of time on the waiting list** |  |  |  |  |  |  |
| 0-6 months | 16 | 16.8 |  |  |  |  |
| 7-12 months | 7 | 7.4 |  |  |  |  |
| 1-2 years | 20 | 21.1 |  |  |  |  |
| 3-5 years | 6 | 6.3 |  |  |  |  |
| 6-10 years | 10 | 10.5 |  |  |  |  |
| 11 years or more | 5 | 5.3 |  |  |  |  |
| **Housing Stress prior to current tenancy** (%yes) | 44 | 46.3 |  |  |  |  |
| **Feels like home** |  |  |  |  |  |  |
| Yes | 57 | 60 |  |  |  |  |
| No | 18 | 18.9 |  |  |  |  |
| **Number of people who regularly (most days of the week) sleep in each bedroom** |  |  |  |  |  |  |
| I person | 60 | 70.6 |  |  |  |  |
| **Number of bathrooms** |  |  |  |  |  |  |
| 1 | 62 | 65.2 |  |  |  |  |
| 2 | 20 | 21.1 |  |  |  |  |
| 3 | * | * |  |  |  |  |
| **Number of toilets** |  |  |  |  |  |  |
| 1 | 57 | 60 |  |  |  |  |
| 2 | 28 | 29.5 |  |  |  |  |
| **Number of cars, vans, trucks parked** |  |  |  |  |  |  |
| 0 | 20 | 21.1 |  |  |  |  |
| 1 | 38 | 40 |  |  |  |  |
| 2 | 20 | 21.1 |  |  |  |  |
| 3 | * | * |  |  |  |  |
| 4 | * | * |  |  |  |  |
| 6 | * | * |  |  |  |  |
| **Utilities/appliances available** |  |  |  |  |  |  |
| Stove/oven/cooking facilities | 72 | 75.8 |  |  |  |  |
| Fridge | 67 | 70.5 |  |  |  |  |
| Heater/heating | 65 | 68.4 |  |  |  |  |
| Washing machine | 65 | 68.4 |  |  |  |  |
| Laundry tub | 58 | 61.1 |  |  |  |  |
| Smoke detectors | 64 | 67.4 |  |  |  |  |
| A landline telephone | 16 | 16.8 |  |  |  |  |
| A computer | 38 | 40 |  |  |  |  |
| An internet connection | 44 | 46.3 |  |  |  |  |
| A television | 62 | 65.2 |  |  |  |  |
| All of the above | 25 | 26.3 |  |  |  |  |
| **In working order** |  |  |  |  |  |  |
| Yes | 55 | 57.9 |  |  |  |  |
| No | 20 | 21.1 |  |  |  |  |
| **Household needs changed** |  |  |  |  |  |  |
| Yes | 23 | 24.2 |  |  |  |  |
| No | 48 | 50.5 |  |  |  |  |
| **Adequacy of housing for needs – living space** |  |  |  |  |  |  |
| Much less than adequate | 15 | 15.8 |  |  |  |  |
| Less than adequate | * | * |  |  |  |  |
| Adequate | 46 | 48.4 |  |  |  |  |
| More than adequate | 12 | 12.6 |  |  |  |  |
| Much more than adequate | * | * |  |  |  |  |
| **Adequacy of housing for needs – bedrooms** |  |  |  |  |  |  |
| Much less than adequate | 12 | 12.6 |  |  |  |  |
| Less than adequate | 10 | 10.5 |  |  |  |  |
| Adequate | 44 | 46.3 |  |  |  |  |
| More than adequate | 10 | 10.5 |  |  |  |  |
| Much more than adequate | 6 | 6.3 |  |  |  |  |
| **Adequacy of housing for needs – distance from public transport** |  |  |  |  |  |  |
| Much less than adequate | 8 | 8.4 |  |  |  |  |
| Less than adequate | * | * |  |  |  |  |
| Adequate | 45 | 47.3 |  |  |  |  |
| More than adequate | 20 | 21.1 |  |  |  |  |
| Much more than adequate | 6 | 6.3 |  |  |  |  |
| **Adequacy of housing for needs – in general** |  |  |  |  |  |  |
| Much less than adequate | 7 | 7.4 |  |  |  |  |
| Less than adequate | 3 | 3.2 |  |  |  |  |
| Adequate | 45 | 47.3 |  |  |  |  |
| More than adequate | 18 | 19 |  |  |  |  |
| Much more than adequate | 6 | 6.3 |  |  |  |  |
| **Prediction of changed housing needs** |  |  |  |  |  |  |
| In the next few weeks | * | * |  |  |  |  |
| In the next six months | * | * |  |  |  |  |
| In the next year | 5 | 5.3 |  |  |  |  |
| In the next 5 years | * | * |  |  |  |  |
| In the next 10 years | * | * |  |  |  |  |
| No change | 25 | 26.3 |  |  |  |  |
| **Absent household members** |  |  |  |  |  |  |
| Yes | 5 | 5.3 |  |  |  |  |
| No | 73 | 76.8 |  |  |  |  |
| **Out of home care** |  |  |  |  |  |  |
| Removed from family and/or child protection services |  |  |  |  |  |  |
| Yes | 16 | 16.8 |  |  |  |  |
| No | 69 | 72.6 |  |  |  |  |
| **Age when first moved out of home** |  |  |  |  |  |  |
| 10-14 | * | * |  |  |  |  |
| 15-19 | 36 | 37.9 |  |  |  |  |
| 20-24 | 10 | 10.5 |  |  |  |  |
| 25-30 | * | * |  |  |  |  |
| 30-34 | * | * |  |  |  |  |
| 35-39 | * | * |  |  |  |  |
| Still at home | 10 | 10.5 |  |  |  |  |
| **Relatives removed from family by government** |  |  |  |  |  |  |
| Yes | 39 | 41.1 |  |  |  |  |
| No | 38 | 40.0 |  |  |  |  |
| **Cultural support plan in place for kinship carers** | * | * |  |  |  |  |
| **Actively support cultural support plan** | * | * |  |  |  |  |
| **CULTURAL SAFETY** |  |  |  |  |  |  |
| **Been treated unfairly in last 12 months** |  |  |  |  |  |  |
| Always | * | * |  |  |  |  |
| Often | 10 | 10.5 |  |  |  |  |
| Sometimes | 20 | 21.1 |  |  |  |  |
| Rarely | 21 | 22.1 |  |  |  |  |
| Only happened once | * | * |  |  |  |  |
| Never | 33 | 34.7 |  |  |  |  |
| **Situations in which treated unfairly** |  |  |  |  |  |  |
| By members of the public | 13 | 13.7 |  |  |  |  |
| Applying for work, or at work | 9 | 9.5 |  |  |  |  |
| Social media | 9 | 9.5 |  |  |  |  |
| By doctors, nurses or other staff at hospitals or doctor’s surgeries | 7 | 7.4 |  |  |  |  |
| By the police, security people, lawyers or in a court of law | * | * |  |  |  |  |
| At home, by neighbours or at somebody else’s house | * | * |  |  |  |  |
| While doing any sporting recreational or leisure activities | * | * |  |  |  |  |
| On the internet or telephone | * | * |  |  |  |  |
| Community service providers | * | * |  |  |  |  |
| When accessing government services | * | * |  |  |  |  |
| At school, university, training course or other educational setting | * | * |  |  |  |  |
| **Experienced in the last 12 months** |  |  |  |  |  |  |
| Heard racial comments or jokes | 23 | 24.2 |  |  |  |  |
| Followed by security while shopping or at a shopping centre | 9 | 9.5 |  |  |  |  |
| Not trusted | 7 | 7.4 |  |  |  |  |
| Left out, refused entry or told you don’t belong | * | * |  |  |  |  |
| Ignored or served last while accessing services or buying something | * | * |  |  |  |  |
| Unfairly questioned by police or arrested or charged | * | * |  |  |  |  |
| Told you were less intelligent | * | * |  |  |  |  |
| Spat at or had something thrown at you | * | * |  |  |  |  |
| I have not had an unfair experience in the last 12 months | 27 | 28.4 |  |  |  |  |
| **Avoided in the last 12 months** |  |  |  |  |  |  |
| Going to public places or events | 7 | 7.4 |  |  |  |  |
| Contact with doctors, nurses or other staff at hospitals or health clinics | 6 | 6.3 |  |  |  |  |
| Contact with police, security people, lawyers or a court of law | 5 | 5.3 |  |  |  |  |
| Applying for work or going to a job | * | * |  |  |  |  |
| Doing any sporting, recreational or leisure activities | * | * |  |  |  |  |
| Seeking other services | * | * |  |  |  |  |
| Contact with government services | * | * |  |  |  |  |
| School, university, training course or other educational setting | * | * |  |  |  |  |
| Other situations | 0 | 0 |  |  |  |  |
| Didn’t avoid any situations | 0 | 0 |  |  |  |  |
| **Culturally safe environments** |  |  |  |  |  |  |
| At home with family | 75 | 79 |  |  |  |  |
| With other Aboriginal and / or Torres Strait Islander people | 43 | 45.3 |  |  |  |  |
| At work | 6 | 6.3 |  |  |  |  |
| At school | * | * |  |  |  |  |
| When playing sport | * | * |  |  |  |  |
| Nowhere | * | * |  |  |  |  |
| Prefer not to answer | 7 | 7.4 |  |  |  |  |
| **Services to support cultural safety** |  |  |  |  |  |  |
| A place to feel culturally safe in this community | 30 | 31.6 |  |  |  |  |
| More cultural events and activities in the community | 27 | 28.4 |  |  |  |  |
| Access to culturally safe employment and training opportunities | 25 | 26.3 |  |  |  |  |
| Access to services such as an Aboriginal Community Controlled Health Organisation | 20 | 21.1 |  |  |  |  |
| Access to schools that celebrate Aboriginal and Torres Strait Islander culture in the curriculum | 19 | 20 |  |  |  |  |
| Access to mainstream service | 10 | 10.5 |  |  |  |  |
| **Aspirations** |  |  |  |  |  |  |
|  |  |  |  |  |  |  |
| **Aspirations** (Response options- not important, slightly important, moderately important, important or very important) | (N= moderately important, important or very important) | (% moderately important, important or very important) | (N=not important and slightly important) | (% not important and slightly important) |  |  |
| Improve my health and wellbeing (this may involve a range of activities to improve your health such as giving up smoking or improving the type of food you eat or activities to help you feel more confident and relaxed) | 66 | 77.70% | 19 | 20.0 |  |  |
| Strengthen relationships with family and friends (this may involve spending more time with family and friend or improving parenting skills) | 62 | 72.9 | 23 | 24.2 |  |  |
| Be involved with culture activities and the local Community (this includes being involved with culturally significant events and local activities run in your community where you meet other people) | 57 | 67.1 | 28 | 29.5 |  |  |
| Own assets (this may involve saving to buy a car or things for your home such as furniture or appliances) | 55 | 64.7 | 30 | 31.6 |  |  |
| Improve my financial situation (this may involve learning how to manage and save money) | 54 | 63.5 | 31 | 32.6 |  |  |
| Develop hobbies, be involved in sports and recreation activities (this might be taking a holiday or joining a sporting club or other activities you enjoy) | 52 | 61.2 | 33 | 34.7 |  |  |
| Improve my education and/or employment (e.g this may involve seeking further skills and training in order to find work in a new area or area of interest) | 47 | 55.30% | 38 | 40.0 |  |  |
| Resources to help me get started with a business (start up and ongoing support, equipment) | 31 | 36.5 | 54 | 56.8 |  |  |
|  |  |  |  |  |  |  |
